# Change in the Distribution Pattern of *Dirofilaria immitis* in Gran Canaria (Hyperendemic Island) between 1994 and 2020

**DOI:** 10.3390/ani14142037

**Published:** 2024-07-11

**Authors:** José Alberto Montoya-Alonso, Sara Nieves García-Rodríguez, Jorge Isidoro Matos, Noelia Costa-Rodríguez, Yaiza Falcón-Cordón, Elena Carretón, Rodrigo Morchón

**Affiliations:** 1 Internal Medicine, Faculty of Veterinary Medicine, Research Institute of Biomedical and Health Sciences (IUIBS), University of Las Palmas de Gran Canaria, 35017 Las Palmas de Gran Canaria, Spain; alberto.montoya@ulpgc.es (J.A.M.-A.); jorge.matos@ulpgc.es (J.I.M.); noelia.costa@ulpgc.es (N.C.-R.); yaiza.falcon@ulpgc.es (Y.F.-C.); elena.carreton@ulpgc.es (E.C.); rmorgar@usal.es (R.M.); 2 Zoonotic Diseases and One Health Group, Faculty of Pharmacy, Biomedical Research Institute of Salamanca (IBSAL), University of Salamanca, 37007 Salamanca, Spain; 3 Centre for Environmental Studies and Rural Dynamization (CEADIR), University of Salamanca, 37007 Salamanca, Spain

**Keywords:** *Dirofilaria immitis*, heartworm disease, zoonosis, vector-borne, prevalence, Gran Canaria, Canary Islands, Spain

## Abstract

**Simple Summary:**

Heartworm disease is a zoonotic illness primarily affecting dogs and cats, which poses a significant risk to public health. Gran Canaria (Canary Island, Spain) serves as a hyperendemic region for this disease, offering a model for its study. This research aimed to track Dirofilariosis prevalence and distribution among dogs, cats, and residents from 1994 to 2020. Data from 5841 dogs, 1203 cats, and 1604 humans were analyzed, considering geographical and climatic factors. Results revealed a decline in *Dirofilaria immitis* prevalence over the years: from 67.02% in dogs (1994) to 15.81% (2020), and from 33.03% (2010) to 17.20% (2020) in cats. Human incidence dropped from 18.66% (2008) to 8.27% (2020). Prevalence was highest in temperate cold zone (TC) and temperate mild zone (TM) climates. Despite a decrease in prevalence, Gran Canaria remains hyperendemic. The study underscores the significance of a “One Health” approach and highlights ongoing risks of disease transmission.

**Abstract:**

Dirofilariosis is a zoonotic disease that mainly affects dogs and cats, with a high risk to public health. The island of Gran Canaria (Spain) has been shown to be a hyperendemic area of infection and, therefore, a model for studying the evolution of the disease. The objective of this study was to track the prevalence and distribution of heartworm in dogs, cats, and residents of Gran Canaria from 1994 to 2020, using published and unpublished data. Blood samples from 5841 dogs, 1203 cats, and 1604 humans were collected in the years analyzed, considering geographical and isoclimatic factors. In 1994, a prevalence of *Dirofilaria immitis* of 67.02% in dogs was reported, while in 2020 it was 15.81%. In cats, the seroprevalence in 2010 was 33.03%, compared to 17.20% in 2020. The incidence of *D. immitis* in humans in 2008 was 18.66%, while in 2020 it was 8.27%. For all study groups, temperate cold zone (TC) and temperate mild zone (TM) climates had the highest prevalence. Throughout these 20 years, the prevalence of heartworm disease has decreased. Despite this, it continues to be a hyperendemic island. This study highlights the importance of using the “One Health” perspective and the risks of contagion of the disease.

## 1. Introduction

*Dirofilaria immitis* is the causative agent of heartworm disease. This pathology mainly affects domestic and wild carnivores (dogs, cats, ferrets, wolves, jackals, among others) [1]. Dogs are considered the main reservoir of the parasite, whereas cats are more resistant to infection. Despite the fact that *D. immitis* is less adapted to cats, their incidence has also been evidenced in regions where there are dogs affected by heartworm disease. Nevertheless, a belief persists that it is being underdiagnosed [2,3]. Furthermore, it is a zoonotic pathogen since humans act as accidental hosts. In them, the parasite cannot complete its life cycle, as they are less susceptible to infection [1]. Globally, the number of infected human cases is increasing, mainly in areas where infected animals are present [4,5,6].

Heartworm disease (dogs and cats) and pulmonary dirofilariosis are transmitted by the bite of culicid mosquitoes of the genus *Culex*, *Aedes*, and *Anopheles*, among others [7,8]. Its distribution is worldwide, being more prevalent in areas with temperate, tropical, and subtropical climates. This is because those areas that have high temperatures and humidity throughout the year favor the proliferation of vectors [9,10]. Furthermore, heartworm cases have been reported more frequently in recent years [11,12,13,14]. Thus, it is an emerging disease that is expanding into areas previously free of the parasite. This problem may be due to the rise in temperatures, the transport of sick animals between endemic countries, the introduction and expansion of new species of vector mosquitoes, irrigated agricultural areas, and human activity in environmental spaces, among others [10].

In the European continent, the disease has spread from the south (traditionally endemic) to north-central Europe, so many of these countries are also considered endemic [10]. Nevertheless, highly variable prevalence can be found within the same region, since they are influenced by orography, climate, and the species of mosquito [14,15,16,17]. In Spain, the continuous expansion of heartworm disease has been demonstrated in recent years, with high prevalence in the south and along the Mediterranean coast, being a hyperendemic disease in both dogs and cats in the Balearic Islands (10.87% prevalence and 16% seroprevalence, respectively) and the Canary Islands (11.58% prevalence and 19.2% seroprevalence, respectively) [18,19].

The Canary Islands have a very similar general climate, characterized by being both a desert and subtropical, moderated by the sea and the trade winds. Despite this, there may be variable climates depending on the geographical region of each island, so different prevalence can be observed between them and their municipalities. In general, El Hierro is considered free of the parasite. On the island of Fuerteventura, only cases of dogs infected with *D. immitis* have been described (prevalence of 1.74%). Meanwhile, cases of dogs and cats exposed to the parasite have been described in Lanzarote (0.99% prevalence and 4.0% seroprevalence, severally). Tenerife, Gran Canaria, La Palma, and La Gomera are considered the islands most affected by *D. immitis* (17.32%, 16.03%, 15.65%, and 11.54%, in dogs, respectively, and seroprevalences of 19.9%, 22.9%, 15.5%, and 29.0% in cats, respectively) [18,19].

Multiple studies have focused mainly on the island of Gran Canaria since 1994, evaluating the high risk of zoonotic infection in Canarian inhabitants, as well as its parasitism in dogs and cats on the island. Multiple variations in the prevalence of these have been observed since the disease began to be studied on the island [20,21,22,23,24,25,26,27,28]. Greater knowledge on the part of society, as well as clinical improvements in the diagnostic, therapeutic, and prophylactic plan, have resulted in a reduction in incidence being achieved. In this way, this hyperendemic island becomes a unique geographical model to identify and investigate the chronological evolution of the parasitism by *D. immitis*.

The aim of this study was to delineate the progression of prevalence and distribution patterns of heartworm in dogs, cats, and inhabitants of Gran Canaria based on data from published studies since 1994, as well as undisclosed information spanning from 2018 to 2020.

## 2. Materials and Methods

### 2.1. Characteristics of Gran Canaria

Gran Canaria is one of the eight volcanic islands of the Canary Islands (Spain), located very close to the African coast of the Sahara (27°37′ and 29°25′ N, 13°20′ and 18°10′ O). It is a circular island that spans a total area of 1560 km^2^, with significant changes in climate, vegetation, and mountainous geography. The highest peak of the island has an altitude of 2000 m, so an evident difference can be observed with respect to the climate that exists on the coast. It is possible to appreciate four isoclimatic zones in Gran Canaria: dry and desert zone (DD), dry and steppe zone (DS), temperate mild zone (TM), and temperate cold zone (TC) [25].

The DD is located from 0 to 200 m, with little rainfall, temperatures above 18 °C (64.4 °F), and very dry summers. The DS ranges from 200 to 500 m, with higher average temperatures than in the DD and longer rainy seasons. Likewise, its atmosphere is cool and pleasant. The TM is between 500 and 1000 m, characterized by dry summers and mild winters. Its temperatures range from 12 °C to 16 °C (53.6–60.8 °F). Finally, the TC goes from 1100 to 2000 m, with very cold winters and temperatures not higher than 22 °C (71.6 °F). There may be temperatures below 0 °C (32 °F) (Köppen Climate Classification, State Meteorological Agency of Spain, 2012).

On the other hand, the population of registered inhabitants on the island of Gran Canaria was 853,252 in 2020 (The Canary Islands Statistics Institute (ISTAC)). Meanwhile, there were 203,917 dogs and 31,758 cats registered in ZOOCAN (Canary Identification Registry).

### 2.2. Samples

On the one hand, annual epidemiological data collected in different years for 3 study groups (dogs, cats, and humans) already published were used. Dogs had data for the years 1994–2002 [20,22], 2007–2010 (except 2009) [22,23,25], and 2015–2020 [26]; cats had data for 2003 [21], 2010 [25], and 2015–2020 [26]; and humans had data for 2008 [23], 2010 [25], 2011 [24], and 2015–2020 [27,28]. On the other hand, samples from dogs and cats between 2018 and 2020 and humans between 2019 and 2020 were analyzed. Therefore, the total number of animals and humans included in this study was 5841 dogs, 1203 cats, and 1604 humans (Table 1). 

Of the serum samples (2018–2020), 404 dogs were analyzed in 2018, 350 in 2019, and 215 in 2020, and for cats, there were 320 analyzed in 2018, 201 in 2019, and 186 in 2020. All animals were examined for routine health checks at veterinary clinics in Gran Canaria. The inclusion criteria in dogs and cats for all years were to be older than 6 months, not having taken prophylaxis against *D. immitis*, and having no previous history of heartworm infection. Identification data (age, breed and sex), clinical history, and demographic data depending on the climate were collected for each animal. In addition, dogs and cats were divided according to their habitat (area where they stayed the longest: outdoors, indoors, mixed). On the one hand, in 2018, 162/404 (40.10%) dogs were indoors, 153/404 (37.87%) were outdoors, and 89/404 (22.03%) were indoors/outdoors. In 2019, 118/350 (33.71%) canine patients were always kept inside the house, 149/350 (42.47%) dogs only had access to the outside, and 83/350 (23.71%) dogs spent at least 1–50% of their time outdoors. Finally, in 2020, 60/215 (27.91%) were indoors, 93/215 (43.26%) were outdoors, and 62/215 (28.84%) were mixed. On the other hand, 132/320 (41.25%) indoor cats were collected in 2018, 104/320 (32.50%) only had access to the outside, and 84/320 (26.25%) cats were indoors/outdoors. In 2019, 56/201 (27.86%) feline patients were kept inside the house, 82/201 (40.80%) lived outdoors, and 63/201 (31.34%) lived both outdoors and indoors. In 2020, 87/186 (46.77%) only had access to the inside of the house, 65/186 (34.95%) outdoor cats were included, and 34/186 (31.34%) mixed cats were collected.

In addition, data were collected from already published human serum samples in 2018 [28], and 163 samples were randomly analyzed in 2019 and 133 in 2020. All samples reflected the demographic data of the human population based on the four isoclimatic zones. 

All human samples were anonymous and had been discarded from a clinical analysis laboratory on the island of Gran Canaria. The confidentiality of the information of the human patients was always maintained. The project was carried out in accordance with current Spanish and European legislation on animal protection. Under Royal Decree 53/2013, these data were extrapolated from clinical veterinary practices for publication after having obtained the owner’s consent.

### 2.3. Study Measurements

For all data already published, different diagnostic methods were used depending on the year of study (Table 1). All canine serum samples from 2018 to 2020 were analyzed using the rapid test for the detection of *D. immitis* antigens (Urano Vet^®^, Barcelona, Spain) according to the manufacturer’s instructions. In the case of the serum samples obtained from feline patients, they were analyzed using three different serological techniques. In 2018 and 2019, commercial kits were used for the detection of specific antibodies against heartworm (HeskaTM Solo StepTM FH, Heska Corporation, Colorado, CO, USA), as well as by indirect Enzyme-Linked ImmunoSorbent Assays (ELISAs) for the detection of anti-*D. immitis* and anti-*Wolbachia* (WSP) antibodies, as previously described [18,21]. Meanwhile, the cat samples obtained in 2020 were tested by an indirect ELISA (in-house ELISA; Urano Vet^®^, Barcelona, Spain) for the detection of specific immunoglobulin G (IgG) antibodies against *D. immitis*, as anteriorly described [29].

Furthermore, all human samples from 2019 to 2020 were analyzed using the ELISA technique for the detection of specific IgG antibodies against *D. immitis*, as previously described [27,30].

### 2.4. Statistical Analyses

Data were analyzed using SPSS Base 26.0 software for Windows (SPSS Inc./IBM, Chicago, IL, USA). For categorical variables, frequencies and percentages were exposed. For continuous variables, the descriptive average, standard deviation, median and interquartile range were shown. The comparisons between parameters were made by general linear models (GLMs) with a powerful estimate (robust covariances) to control the infractions of the assumptions of the model. The marginal stockings and their 95% confidence intervals (CIs) were displayed. All multiple comparisons were adjusted by the correction of Bonferroni. Pearson’s correlation coefficients were also evaluated with the coefficient value and its *p*-value. The significance level used in the analyses was 5% (α = 0.05). 

## 3. Results

### 3.1. Total Prevalence According to the Year in Dogs and Cats

**Figure 1:**
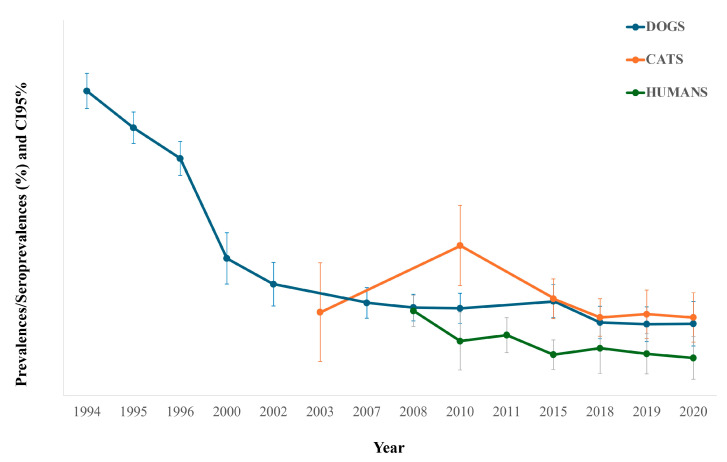
Total prevalence/seroprevalence per year in dogs, cats, and humans studied, with their confidence interval 95% (CI95%). Blue line: dogs’ prevalence; red line: cats’ seroprevalence; green line: humans’ seroprevalence.

The total prevalence by year in dogs and cats is displayed in Figure 1 and Table 2. In relation to dogs, a decrease in prevalence from 67.02% in 1994 [20] to 15.81% in 2020 is observed. The seroprevalence in cats remained between 18.37% (2003) [21] and 17.20% (2020), despite higher seroprevalences between these years, such as 33.03% in 2010 [25] and 21.30% in 2015 [26]. 

### 3.2. Total Prevalence According to the Year in Humans

Figure 1 and Table 2 show the yearly seroprevalence rates in humans. According to this, a decrease is also observed between 2008 (18.66%) [23] and 2020 (8.27%).

### 3.3. Difference in Prevalence between 3 Study Groups in General

#### 3.3.1. Total Prevalence

**Figure 2:**
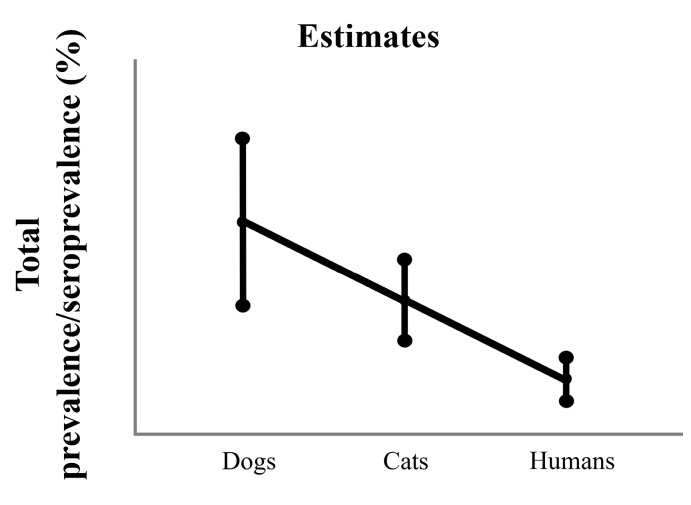
Average prevalence/seroprevalence of the years available for 3 study groups. The graph shows the estimated marginal means and 95% confidence interval.

After averaging the prevalence of the years available for three study groups, the linear model that compares the total prevalence of the disease between three study groups indicated with its *p*-value 0.000 (GML; F(2,22) = 11.170, *p* < 0.001) that there were statistical differences between them. In this way, the total prevalence between dogs and cats was equal, while that of humans was statistically lower than the other two study groups (Figure 2).

#### 3.3.2. According to the Climate Conditions

Significant differences were observed between each isoclimatic zone. In the DD climate, the total prevalence in dogs was more than double that in cats and humans. Also, the total prevalence in dogs was double that in the inhabitants of the TM climate and tripled the prevalence of humans in the DS climate. Likewise, the prevalence in humans was almost one third of that in dogs and more than half of that in cats in the case of the TC climate (Table 3). In general, the prevalences of the four isoclimatic zones were highly correlated with each other, with Pearson’s r coefficients greater than 0.6.

**Figure 3:**
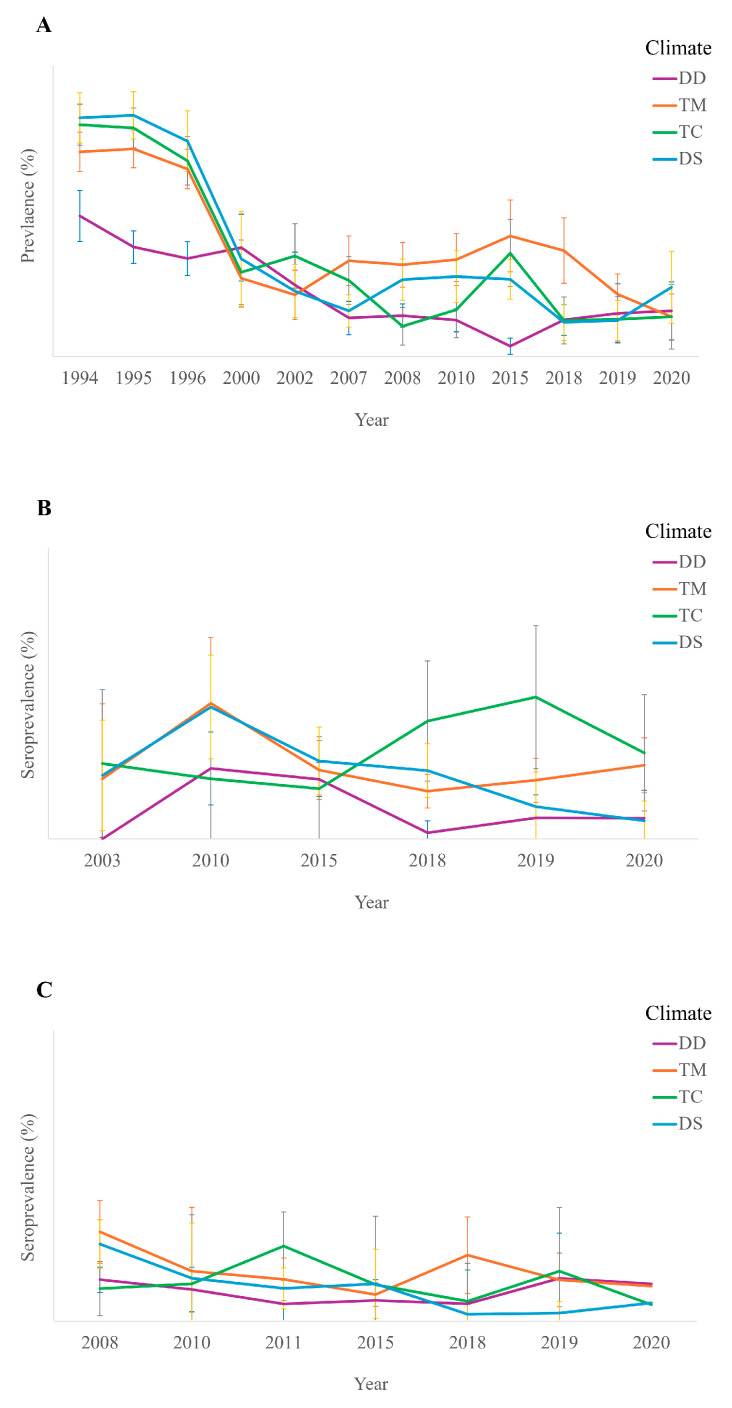
Average prevalence according to year and climatic zone for 3 study groups ((**A**): dogs; (**B**): cats; (**C**): humans), taking into account their confidence interval 95% (CI95%).

On the other hand, the prevalence was studied by climate, three study groups, and year to obtain an average prevalence (Figure 3). In 1994, the prevalence of *D. immitis* in dogs was found to be 46.62% in the DD climate, 79.12% in the DS climate, 67.86% in the TM climate, and 76.87% in the TC climate [20]. Meanwhile, in 2020, the incidence was 15.09% in the DD climate, 22.92% in the DS climate, 13.16% in the TM climate, and 13.19% in the TC climate.

Meanwhile, cats had an incidence in 2003 of 0% in the DD climate, 21.05% in the DS climate, 20% in the TM climate, and 25% in the TC climate [21]. Conversely, in 2020, they had an incidence of 6.9% in the DD, 6% in the DS, 24.42% in the TM, and 28.57% in the TC.

Finally, climate incidence in humans was studied for the first time in 2008, finding a prevalence of 13.85% in the DD climate, 25.66% in the DS climate, 29.73% in the TM climate, and 10.86% in the TC climate [23]. Conversely, in 2020, an incidence of 12.50% was observed in the DD climate, 6.15% in the DS climate, 11.76% in the TM climate, and 5.56% in the TC climate. 

### 3.4. Between 2018 and 2020

The descriptive analysis showed a trend towards stability in the prevalence between 2018 and 2020 in dogs and cats. Meanwhile, the prevalence in humans showed a slight downward trend between 2018 and 2020. Moreover, inhabitants of Gran Canaria presented a lower mean prevalence than dogs and cats, while these last two study groups showed a mean prevalence equal to each other (F(2,6) = 114.196, *p =* 0.000).

Regarding the influence of climate, there was no significant evolution between 2018 and 2020 for the TM and DS areas. In the meantime, there was an increase in prevalence in the DD zone between 2018 and 2019. In the case of the TC climate, there was an increase in 2019 and a decrease in 2020 (GML; F(8,24) = 24.983, *p* < 0.001).

### 3.5. Influence of Habitat on Dogs and Cats between 2018 and 2020

The habitat was not considered in the previous studies already published, unlike between 2018 and 2020. Significant differences were observed in the percentages of positives after analyzing the habitat of dogs and cats as a whole (GML; F(2,9) = 41.172, *p* < 0.0001), with no differences between 2018 and 2020 (GML; F(6,9)=1.346, *p =* 0.330). 

In the case of cats, the percentage of positives outdoors and mixed was the same (without separating by year), while those cats that were kept exclusively inside the house showed a lower percentage compared to the other two habitats (F(2,6) = 12.177, *p* = 0.008). In the same way, the percentage of positives for each habitat (without separating by year) in dogs was the same for those with mixed and outdoor access, and lower for indoor ones (F(2,6) = 88.906, *p* = 0.000).

#### Positives’ Evolution

Between 2018 and 2020, no significant evolution was shown in the percentage of positives in both dogs and cats (F(2,9) = 1.058, *p* = 0.387). Nevertheless, significant differences were observed by habitat (F(3,9) = 17.503, *p* < 0.001), so that those with only indoor access did not see any evolution (*p* = 0.247), contrary to mixed and outdoor habitats (*p* = 0.000 and *p* = 0.004, respectively). That is, those animals that lived in a mixed habitat increased their percentage of positives between 2018 and 2019 (14%), and this was maintained in 2020. In dogs and cats that only had access to the outside, the percentage of positives decreased between 2018 and 2019 (26%) and increased in 2020.

## 4. Discussion

Heartworm disease has a worldwide distribution. Even though the parasite is not considered endemic to certain countries, its manifestation has been published about on all continents [13,14,31,32,33,34,35,36]. The presence of hematophagous mosquito species is required for transmission of the nematode to occur, as well as parasitized dogs that develop microfilariae in blood circulation [1]. Owing to this, isolated cases have been detected worldwide due to the expansion of new vector species, the frequent transport of infected animals to places free of the disease, and trips without prophylaxis to endemic areas [37,38]. Within the European continent, the parasite has been able to spread from the southern Mediterranean regions to the northern and eastern regions [37]. In Italy, the northern region and the Po Valley are considered endemic. However, a gradual increase in the overall prevalence in Italy has been observed in recent years (from 0.77% in 2009 to 8.47% in 2016–2017) [17]. 

In the same way, Spain has proven to be a country that presents favorable climatic conditions for the expansion of *D. immitis*. In 2022, a total prevalence of 6.47% was detected, and it was observed that all the autonomous communities and cities showed cases of infected dogs. In addition, the parasite was found in provinces and islands that were considered free of the disease [19].

Gran Canaria is an island with a very high risk of infection [39]. According to Morchón et al., 2023 [40], Gran Canaria has ideal climatic conditions. For this reason, it is considered an example to study heartworm disease. Based on all the data collected (*n* = 5841 + 1203 + 1604) through previously published and current studies, it has been observed that the positive cases for *D. immitis* in three study groups have varied over the years.

The results indicate that there has been a general downward trend, until its current stabilization. In the case of the feline group, there was a rise in seroprevalence from 2003 (18.37%) [21] to 2010 (33.03%) [25]. This may be due to the difference in the sample number (49 cats in 2003 and 109 cats in 2010), as well as the diagnostic test that was different for the two years. In both years, the ELISA technique for the detection of specific antibodies against *D. immitis* was used, but with some modifications in the processing and the cut-off for both cases [21,25]. Meanwhile, in the years analyzed for dogs and humans, a generally decreasing graph has been observed. Epidemiological studies carried out in 1994 found a prevalence of *D. immitis* of 67.02% in the canine population [20], whereas the results in 2020 revealed an incidence of 15.81%. By the same token, the incidence of *D. immitis* in humans in 2008 was 18.66% [23], while in 2020, it was 8.27%. Likewise, there was a significantly higher overall prevalence in dogs and humans in 2008 [23] than between 2018 and 2020, probably because the population has generated greater awareness and responsibility for prophylaxis against the disease.

Based on the results obtained, it was observed that there has been a stabilization in the prevalence of cases in dogs and seroprevalence in cats between 2018 and 2020, while a slight downward trend continues in the total prevalence of humans. Furthermore, the prevalence of humans was lower, probably because these are accidental hosts.

On the other hand, other studies have observed that the Canary Islands have a higher risk of infection in coastal areas with humidity and a human footprint [40]. Through these results, it was noticed that the total prevalence by climate was lower in the DD areas during the first years studied. However, an increase was observed between 2018 and 2019. Over the years, it has been observed that the four climates have generally correlated with each other. Climate change is an important factor to take into account after these results, since high temperatures favor the proliferation of the transmitting vector, as well as humidity and water availability.

The habitat was not analyzed in the published studies, so this study includes the incidences of dogs and cats between 2018 and 2020, according to the area where they spend the most time. It was observed that the percentage of positives in dogs and cats studied between 2018 and 2020 (without separating by year) was lower in those that had exclusively indoor access. Even though the spread of this disease occurs through mosquito bites, previous studies have shown that animals that live indoors are less exposed to the presence of vector mosquitoes and, therefore, the prevalence is lower. However, the need to use chemoprophylactic methods is not exempted since mosquitoes can be found inside homes. Furthermore, the appearance and expansion of new species of invasive mosquitoes with the capacity to act as vectors may present diurnal feeding habits, further increasing the risk of infection in these animals. Likewise, those animals with a mixed habitat showed an increase in the percentage of positives between 2018 and 2019, to subsequently maintain itself in 2020. Meanwhile, patients with exclusively outside access showed a reduction in the percentage of positives between 2018 and 2019, and they increased in 2020. 

These results demonstrate the successful implementation of control and prophylaxis measures. The stability observed since 2015 is probably due to the persistence of untreated reservoirs (mainly dogs used for hunting and kept in poor hygienic–sanitary conditions). In addition, the presence of vector mosquitoes and the introduction of new competent vector species should be considered. Therefore, the collaboration of veterinarians and owners is required to control the incidence of heartworm disease. The results also show the zoonotic importance of this disease and, therefore, the need for surveillance and control measures.

## 5. Conclusions

Through this study, it has been possible to determine the incidence and the chronological evolution of the disease in dogs, cats, and humans over the last 20 years on the island of Gran Canaria. This study highlights the importance of the concept “One health”, since all healthcare professionals should be aware of the contagion risk and the status of this disease to consider what the future may hold for us. Taking into account the high risk of infection on this island and the variability in climates, the implementation of preventive and awareness campaigns would be ideal to further decrease the prevalence of the disease.

## Figures and Tables

**Table 1:** Number of samples per study group for each year based on published and unpublished data. The diagnostic method used for each study group is detailed according to the year. * Vetred Dirofilaria, Rhône Mérieux; ** Canine Heartworm Antigen, IDEXX Laboratories Inc. (Barcelona, Spain); *** Urano test Dirofilaria^®^, Urano Vet SL, Barcelona, Spain.

Year	Dogs	Cats	Humans	Diagnostic Method
**1994**	567 [20]			**Dogs**: Haemagglutination test *
**1995**	779 [20]			**Dogs**: Haemagglutination test *
**1996**	688 [20]			**Dogs**: Haemagglutination test *
**2000**	255 [22]			**Dogs**: Commercial ELISA test kit **
**2002**	310 [22]			**Dogs**: Commercial ELISA test kit **
**2003**		49 [21]		**Cats**: Non-commercial ELISA technique
**2007**	551 [22]			**Dogs**: Commercial ELISA test kit **
**2008**	697 [23]		493 [23]	**Dogs**: Commercial ELISA test kit ** **Humans**: Non-commercial ELISA (antibodies) technique
**2010**	547 [25]	109 [25]	100 [25]	**Dogs**: Commercial ELISA test kit ** **Cats**: Non-commercial ELISA (antibodies) technique **Humans**: Non-commercial ELISA (antibodies) technique
**2011**			300 [24]	**Humans**: Non-commercial ELISA (antibodies) technique
**2015**	478 [26]	338 [26]	300 [27]	**Dogs**: Commercial immunochromatographic test kit *** **Cats**: Non-commercial ELISA (antibodies) technique **Humans**: Non-commercial ELISA (antibodies) technique
**2018**	404	320	115 [28]	**Dogs**: Commercial immunochromatographic test kit *** **Cats**: Non-commercial ELISA (antibodies) technique **Humans**: Non-commercial ELISA (antibodies) technique
**2019**	350	201	163	**Dogs**: Commercial immunochromatographic test kit *** **Cats**: Non-commercial ELISA (antibodies) technique **Humans**: Non-commercial ELISA (antibodies) technique
**2020**	215	186	133	**Dogs**: Commercial immunochromatographic test kit *** **Cats**: Non-commercial ELISA (antibodies) technique **Humans**: Non-commercial ELISA (antibodies) technique

**Table 2:** Total prevalence of dogs and seroprevalence of cats and humans over the years.

Year	Dogs’ Prevalence	Cats’ Seroprevalence	Humans’ Seroprevalence
**1994**	67.02% [20]		
**1995**	58.92% [20]		
**1996**	52.18% [20]		
**2000**	30.19% [22]		
**2002**	24.50% [22]		
**2003**		18.37% [21]	
**2007**	20.40% [22]		
**2008**	19.36% [23]		18.66% [23]
**2010**	19.20% [25]	33.03% [25]	12% [25]
**2011**			13.3% [24]
**2015**	20.77% [26]	21.30% [26]	9% [27]
**2018**	16.09%	17.19%	10.43% [28]
**2019**	15.71%	17.91%	9.2%
**2020**	15.81%	17.20%	8.27%

**Table 3:** Total prevalence in dogs and seroprevalence in cats and humans depending on the isoclimatic zone. ^+^ Mean ± SD; *p* = *p*-value GLM.

	Dogs	Cats	Humans	
**DD Climate positives ^+^**	36.00 ± 33.92 (21.53 ± 13.23)	9.17 ± 14.66 (9.85 ± 9.56)	8.14 ± 12.43 (9.99 ± 3.73)	*p* = 0.023
**TM Climate positives ^+^**	54.92 ± 50.09 (37.36 ± 18.94)	16.00 ± 9.01 (24.60 ± 10.42)	10.86 ± 7.27 (16.69 ± 7.05)	*p* = 0.005
**TC Climate positives ^+^**	38.25 ± 43.06 (33.44 ± 25.15)	5.17 ± 2.93 (29.41 ± 11.64)	3.57 ± 4.83 (12.78 ± 6.56)	*p* = 0.002
**DS Climate positives ^+^**	33.83 ± 24.42 (35.29 ± 25.88)	9.67 ± 7.15 (21.67 ± 13.21)	7.29 ± 9.93 (10.68 ± 8.09)	*p* = 0.008

## Data Availability

The raw data supporting the conclusions of this article will be made available by the authors without undue reservation.
